# Impact of perceived control on all-cause and cardiovascular disease mortality in three urban populations of Central and Eastern Europe: the HAPIEE study

**DOI:** 10.1136/jech-2017-208992

**Published:** 2017-05-17

**Authors:** Magdalena Kozela, Andrzej Pająk, Agnieszka Micek, Agnieszka Besala, Ruzena Kubinova, Sofia Malyutina, Abdonas Tamosiunas, Hynek Pikhart, Anne Peasey, Yuri Nikitin, Michael Marmot, Martin Bobak

**Affiliations:** 1 Chair of Epidemiology and Population Studies, Faculty of Health Sciences, Jagiellonian University Medical College, Krakow, Poland; 2 National Institute of Public Health, Prague, Czech Republic; 3 Institute of Internal and Preventive Medicine, Novosibirsk, Russia; 4 Novosibirsk State Medical University, Novosibirsk, Russia; 5 Institute of Cardiology, Lithuanian University of Health Sciences, Kaunas, Lithuania; 6 Department of Epidemiology and Public Health, University College London, London, UK

**Keywords:** Cardiovascular disease, MORTALITY, PSYCHOSOCIAL FACTORS, EASTERN EUROPE

## Abstract

**Background:**

Inverse associations between perceived control and cardiovascular disease (CVD) have been reported in studies from Western Europe and the USA. To assess this relationship across different populations, we investigated the association between perceived control and all-cause and CVD mortality in three population-based cohorts of Eastern European countries.

**Methods:**

We analysed data from a prospective cohort study in random population samples in Krakow (Poland), Novosibirsk (Russia) and six Czech towns. Baseline survey included structured questionnaire and objective examination in a clinic. Perceived control was assessed using an 11-item scale developed by the MacArthur Foundation Programme on Successful Midlife. Information on vital status was obtained from death registers. Effect of perceived control on mortality was assessed using Cox proportional hazards models.

**Results:**

A total of 2377 deaths (1003 from CVD) occurred among 27 249 participants over a median 7-year follow-up. In the Czech and Polish cohorts, perceived control was inversely associated with mortality; the adjusted HRs for the lowest versus highest control quintiles were 1.71 (1.34 to 2.19) in men and 1.63 (1.14 to 2.35) in women for all-cause mortality and 2.31 (1.48 to 3.59) and 5.50 (2.14 to 14.13) for CVD deaths. There was no association between perceived control and mortality in Russia; the adjusted HRs for all-cause mortality were 1.03 (0.79 to 1.34) in men and 1.29 (0.82 to 2.02) in women.

**Conclusions:**

Low perceived control was associated with increased risk of all-cause and CVD mortality in Czech and Polish cohorts but not in Russia. It is possible that this inconsistency may partly reflect a different sociocultural understanding of the concept of control in Russia.

## Introduction

Psychosocial and psychological factors, such as anxiety, depression, social networks and support as well as work stress, have been shown to be associated with cardiovascular disease (CVD) mortality and incidence.^1–5^ The mechanisms linking psychosocial factors with CVD are not fully understood, and are likely to involve both direct pathways (eg, metabolic changes resulting from neuroendocrine response, imbalances of the sympathetic and parasympathetic nervous system or neuroimmunological disturbances^6–8^) or indirect pathways via health behaviours, such as smoking, alcohol consumption, physical activity or diet.^9^ ^10^

Perceived control, often defined as a belief that one is able to determine his/her own behaviour, influence his/her environment and attain desired outcomes,^11^ can help individuals to deal with stressors in their lives. Perceived control is considered to be related to the constructs of locus of control, self-efficacy and mastery,^12^ and it often refers to the perceived ease or difficulty to influence the desired outcomes of own actions and behaviours.^11^ ^13^ ^14^ For example, poverty or unemployment is related to low perceived control, but re-employment was found to be related with higher perceived control levels.^15^ ^16^ Inverse associations of perceived control and related constructs with CVD have been reported from several case–control and cohort studies.^17–23^ As existing evidence on the relationship between perceived control and health relies on observational studies, the causality of the association remains debated.

Virtually all existing evidence on the relationship between perceived control and health comes from Western Europe and the USA. These are stable, high-income and relatively individualistic societies; confirmation studies in non-Western societies would be to assess the consistency of the association across different populations. Central and Eastern Europe seems a particularly interesting setting, since this region represents a variety of historically determined social and cultural characteristics that might influence the perception of self-control in individuals. For example, in the communist system, the perception of individual control over one's life was probably low. Similarly, the turbulent political transformation, followed by significant social and economic changes, is also likely to have influenced psychological and psychosocial well-being.

The objective of the present study was to investigate the association between perceived control and all-cause and CVD mortality in three Eastern European, population-based cohorts and to assess the consistency of the associations between populations. Given the differences in sociopolitical environment between Eastern and Western Europe, the postcommunist societal transformation high CVD death rates in Eastern Europe, and different patterns of potential confounding factors in different societies, studying the association of perceived control with CVD in this setting can provide useful evidence to either support or refute the hypothesis that perceived control is an important predictor of CVD.

## Methods

### Study populations and subjects

The present analysis used data on the 28 945 participants in the Health Alcohol and Psychosocial factors In Eastern Europe (HAPIEE) prospective cohort study in Krakow (Poland), Novosibirsk (Russia) and six towns of the Czech Republic (Havírov/Karvina, Hradec Kralove, Jihlava, Krome, Liberec and Ustı nad Labem). Random samples of male and female residents aged 45–69 years were examined during 2002–2005. The overall response rate was 59% (61% in Krakow and Russia and 55% in the Czech towns). The study was approved by ethical committees in all participating centres and at University College London. All participants gave informed consent for attendance in the study.

### Data collection

Detailed description of the HAPIEE study size and protocol was described previously;^24^ information specific to this paper is given below. Baseline information on participants was collected by structured questionnaire and objective examination in a clinic which included a blood sample.

### Measurements

Perceived control was assessed using an 11-item scale which was initially developed by the MacArthur Foundation Programme on Successful Midlife^25^ and subsequently used in the Whitehall II study and by the New Democracy Barometer surveys.^18^ ^26^ Participants indicated the extent to which they agreed or disagreed with 11 statements referring to their perceived control over life events and health; the responses were recorded on a 6-point scale (ranging from totally agree to totally disagree, coded from 0 to 5). The sum of these responses ranged from 0 (total lack of control) to 55 (maximum perceived control). The algorithm allowed at most two missing answers (replaced by the arithmetic mean of valid responses). Participants were then classified into five sex-specific quintiles.

The following covariates measured at baseline were used in the analysis. Participants were classified into one of four categories of their highest achieved education (primary or lower, vocational, high, university). Marital status was dichotomised as married/cohabiting versus single/divorced/widowed. Two categories of occupational status were defined: working (employees, entrepreneurs, freelancers, farmers, working pensioners) and non-working (housewives, pensioners, unemployed). Persons declaring smoking cigarettes regularly or occasionally were classified as current smokers; ex-smokers were those who had smoked in the past but stopped; and persons who had never smoked cigarettes were considered as never smokers. Alcohol intake was assessed using Graduated Frequency Questionnaire and expressed in grams of pure ethanol per year. Positive history of CVD (myocardial infarction, angina or stroke) or cancer was based on self-report. Hypertension was defined as measured blood pressure ≥140/90 mm Hg or using antihypertensive medication in the past 2 weeks. Hypercholesterolaemia was defined as total cholesterol ≥5 mmol/L or LDL-C ≥3 mmol/L or using lipid-lowering medication. Diabetes was defined as having fasting plasma glucose ≥7 mmol/L or having diabetes diagnosed by doctor.

### Mortality follow-up

Cause-specific mortality in the cohorts was recorded during subsequent 7–8 years after the baseline examination (median time of 7 years in Krakow and Novosibirsk and 8 years in the Czech cohort), using the following registers: the City of Krakow register and Central Statistical Office in Poland; mortality register developed by the Institute of Internal Medicine based on data from the Novosibirsk office of the State Statistical Bureau (Goskomstat) and from the population registration bureau; and National Death Register in the Czech Republic. Classification of causes of deaths was made using the International Statistical Classification of Diseases and Related Health Problems—Review X (ICD-10); CVD deaths were those with codes I.00–I.99.

### Statistical analyses

Out of 28 945 persons examined at baseline, 27 702 (96%) had follow-up data available. Further, 453 were excluded due to missing data on perceived control, leaving 27 249 persons (191 967 person-years) in the analysis. Descriptive characteristics were tabulated by study centre and in the pooled sample for men and women separately. Significance tests for differences in distributions of participants' characteristics between study centres were calculated using χ^2^ test, ANOVA or Kruskal-Wallis test depending on the type of the variable and its distribution. Association between perceived control quintile and all-cause and CVD mortality was assessed using Cox proportional hazards regression models, with the highest sex-specific quintile (very high control) as reference category. The assumptions of proportional hazards were checked using Schoenfeld's test examining interactions with time to the event. Analyses were performed separately for each centre.

Since the relationship between perceived control and mortality in Russia differed from the Czech and Polish cohorts (in which the associations were similar), with highly significant interaction term in men (p<0.001), we were unable to pool the data from all three cohorts but we could combine the Polish and Czech data. Although in women the interaction term was not statistically significant, probably because of lower statistical power due to smaller number of deaths, the main analyses in both sexes present the Russia cohort separately from the pooled Czech and Polish cohorts. Three models were fitted: model 1 was adjusted for age and cohort (in case of the pooled Czech and Polish data); model 2 additionally adjusted for education, marital status, occupation, history of CVD and history of cancer (only for analyses of all-cause mortality); and model 3 further adjusted for smoking, hypertension, hypercholesterolaemia, diabetes and alcohol consumption. Physical activity and body mass index were not included into the multivariate models because they did not alter the estimated HRs. Owing to the two-stage character of the examination (health questionnaire at home and physical examination in clinics in Poland and Czech Republic), the participation rate for the clinical examination was lower than for the interview. As a consequence, the number of persons included in the model 3 was lower (by approximately 15%) than in model 1 and 2 as the sample was restricted to participants with all complete records in all covariates. Statistical analyses were conducted in R V.3.02 (R Foundation for Statistical Computing, Vienna, Austria) and STATA V.12.1 (StataCorp LP, Texas, USA).

## Results

Table 1 presents descriptive statistics of study participants by sex and country. At baseline, total number of 13 617 men and 15 328 women were examined. Out of them 812 (5.96%) men and 884 (5.76%) women were excluded due to lack of follow-up information or data on perceived control. Finally, 88 252.35 and 103 715.30 person-years were analysed in men and women, respectively. In men, the highest proportion of deaths was observed in Russia (n=627, 15.1%), compared with Poland (n=530, 11.1%) and Czech Republic (n=439, 11.5%). Also in Russia, CVD deaths accounted for over 50% of all deaths, while in Poland and Czech Republic for <40%.

**Table 1:** Sample size, number of deaths and distribution of covariates by country and sex

	Czech Republic		Poland		Russia		Total	
*Men*								
Baseline examination	3830		4789		4186		12 805	
Person-years	30 176		33 002		25 075		88 252	
Number of deaths	439	11.5	530	11.1	627	15.1	1596	12.5
Number of CVD deaths	168	4.4	184	3.8	339	8.2	691	5.4
Follow-up median (IQR)	8.1	(7.7–8.9)	7.1	(6.8–7.7)	6.2	(5.78–6.9)	7.1	(6.5–7.8)
Perceived control (x, SD)	35.5	7.28	37.1	7.47	34.5	8.0	35.8	7.67
Age (x, SD)	58.5	7.16	57.9	6.94	58.3	7.04	58.2	7.04
Education (n, %)								
Primary	223	5.8	436	9.1	476	11.4	1135	8.9
Vocational	1663	43.7	1307	27.3	913	21.8	3883	30.4
High	1219	32.0	1599	33.4	1455	34.7	4273	33.4
University	704	18.5	1444	30.2	1342	32.1	3490	27.3
Marital status (n, %)								
Married/cohabiting	3211	84.2	4100	86.8	3681	87.9	11 039	86.4
Single/divorced/widowed	603	15.8	629	13.2	505	12.1	1737	13.6
Occupational status (n, %)								
Working	2215	58.1	2324	48.6	2579	61.6	7118	55.7
Not working	1595	41.9	2458	51.4	1607	38.4	5660	44.3
Smoking (n, %)								
Current smoker	1118	29.5	1721	36.0	2074	49.6	4913	38.5
Ex-smoker	1459	38.5	1729	36.2	1036	24.8	4224	33.1
Never smoker	1212	32.0	1326	27.8	1076	25.7	3614	28.3
Hypercholesterolaemia (n, %)	2580	78.6	3619	81.8	3506	84.0	9705	81.7
Hypertension (n, %)	2303	72.6	2782	66.3	2646	63.3	7731	67.0
Diabetes (n, %)	768	20.1	775	16.2	450	11.1	1993	15.7
History of CVD (n, %)	575	15.6	1115	23.6	987	23.6	2677	21.3
History of cancer (n, %)	156	4.2	156	3.3	54	1.3	366	2.9
Alcohol intake (g/year), Me (Q1–Q3)	3120	(512.5–9950)	800	(40–3125)	2455	(480–7800)	1820	(270–6105)
*Women*								
Baseline examination	4350		5066		5028		14 444	
Person-years	35 213		35 967		32 535		103 715	
Number of deaths	242	5.6	283	5.6	256	5.1	781	5.4
Number of CVD deaths	81	1.9	95	1.9	136	2.7	312	2.2
Follow-up Me (Q1–Q3)	8.2	(7.77–8.87)	7.1	(6.86–7.75)	6.7	(5.97–7.10)	7.1	(6.75–7.85)
Perceived control (x, SD)	34.3	7.54	36.3	7.54	33.6	7.94	34.7	7.77
Age (x, SD)	58.0	7.10	57.4	6.98	58.0	7.13	57.8	7.08
Education (n, %)								
Primary	771	17.8	670	13.2	478	9.5	1919	13.3
Vocational	1332	30.7	765	15.1	1541	30.7	3638	25.2
High	1791	41.3	2250	44.5	1681	33.4	5722	39.7
University	441	10.2	1377	27.2	1328	26.4	3146	21.8
Marital status (n, %)								
Married/cohabiting	2956	68.2	3376	66.8	2990	59.5	9322	64.7
Single/divorced/widowed	1379	31.8	1681	33.2	2038	40.5	5098	35.4
Occupational status (n, %)								
Working	2047	47.3	1999	39.5	2359	46.9	6405	44.4
Not working	2285	52.8	3060	60.5	2669	53.1	8014	55.6
Smoking (n, %)								
Current smoker	1019	23.6	1454	28.8	515	10.2	2988	20.8
Ex-smoker	952	22.1	1061	21.0	217	4.3	2230	15.5
Never smoker	2340	54.3	2541	50.3	4296	85.4	9177	63.8
Hypercholesterolaemia (n, %)	3097	81.2	3997	85.0	4571	91.3	11 665	86.3
Hypertension (n, %)	2174	58.3	2484	55.8	3359	66.9	8017	60.7
Diabetes (n, %)	606	13.9	588	11.6	566	11.5	1760	12.3
History of CVD (n, %)	402	9.8	1051	21.0	1006	20.0	2459	17.4
History of cancer (n, %)	342	8.2	312	6.2	200	4.00	854	6.0
Alcohol intake (g/year), Me (Q1–Q3)	300	(40–1420)	40	(0–360)	270	(40–433)	120	(0–630)

CVD, cardiovascular disease; x, mean.

Perceived control was highest in Poland and only slightly lower in the Czech Republic and Russia. There were differences in educational achievements between countries, with higher levels in the Polish and Russian cohorts compared with the Czech participants. Over four-fifths of men and two-thirds of women were married or cohabiting. Smoking was most common among Russian men and least common among Russian women. There were also differences in the prevalence of high cholesterol, hypertension and the history of CVD and diabetes between cohorts. Differences in all covariates between cohorts were statistically significant (p<0.001) (table 1).

Tables 2 and 3 show the relationships between perceived control and total and CVD mortality by each cohort separately and in the combined Polish and Czech samples. In analyses of all-cause mortality (table 2), there was a significant inverse association in Czech and Polish men and women. In the age-adjusted model, men and women with very low perceived control had more than 2.5 times higher risk of death than those with very high control, and the inverse trend was statistically significant. Adjustment for education, marital status, occupational status, history of CVD and history of cancer reduced the HRs by about one-third, and additional adjustment for smoking, hypertension, hypercholesterolaemia, diabetes and alcohol consumption further attenuated the estimates of HRs to 1.71 (1.34 to 2.19) and 1.63 (1.14 to 2.35), respectively. In Russia, in contrast, the association was considerably weaker; the age-adjusted HR of men and women in the lowest versus highest control groups were 1.35 (1.05 to 1.75) and 1.58 (1.01 to 2.47), respectively, and after adjustment for all covariates the estimates became weak or non-existent and no longer statistically significant (table 2).

**Table 2:** Association between perceived control and risk of death from all causes

	Men						Women					
	Czech Republic	Poland	p Value for interaction (CZ × PL)	Czech Republic and Poland	p Value for interaction (3 countries)	Russia	Czech Republic	Poland	p Value for interaction (2 countries)	Czech Republic and Poland	p Value for interaction (3 countries)	Russia
	HR (95% CI)	HR (95% CI)		HR (95% CI)		HR (95% CI)	HR (95% CI)	HR (95% CI)		HR (95% CI)		HR (95% CI)
*Model 1*												
Very high	1.00	1.00	0.5824	1.00	0.0008	1.00	1.00	1.00	0.8871	1.00	0.5814	1.00
High	1.27 (0.85 to 1.89)	1.16 (0.86 to 1.57)		1.20 (0.94 to 1.52)		1.00 (0.73 to 1.36)	1.16 (0.66 to 2.05)	1.45 (0.96 to 2.19)		1.34 (0.96 to 1.87)		0.97 (0.57 to 1.62)
Moderate	1.58 (1.09 to 2.30)	1.44 (1.08 to 1.93)		1.50 (1.19 to 1.88)		1.16 (0.88 to 1.52)	1.32 (0.74 to 2.33)	1.30 (0.84 to 2.01)		1.31 (0.93 to 1.85)		1.04 (0.64 to 1.68)
Low	2.15 (1.50 to 3.09)	1.75 (1.32 to 2.32)		1.91 (1.53 to 2.38)		1.17 (0.90 to 1.52)	1.58 (0.93 to 2.70)	1.88 (1.24 to 2.84)		1.75 (1.26 to 2.43)		0.98 (0.61 to 1.58)
Very low	2.58 (1.82 to 3.65)	2.79 (2.12 to 3.67)		2.65 (2.14 to 3.29)		1.35 (1.05 to 1.75)	2.68 (1.62 to 4.44)	2.72 (1.82 to 4.06)		2.75 (2.02 to 3.76)		1.58 (1.01 to 2.47)
p Value for trend	<0.001	<0.001		<0.001		0.0071	<0.001	<0.001		<0.001		0.0061
*Model 2*												
Very high	1.00	1.00	0.5008	1.00	0.0025	1.00	1.00	1.00	0.8520	1.00	0.6361	1.00
High	1.10 (0.73 to 1.66)	1.10 (0.81 to 1.50)		1.10 (0.86 to 1.40)		0.97 (0.72 to 1.32)	1.29 (0.70 to 2.37)	1.30 (0.86 to 1.97)		1.29 (0.91 to 1.81)		0.95 (0.57 to 1.60)
Moderate	1.29 (0.88 to 1.90)	1.24 (0.92 to 1.67)		1.27 (1.00 to 1.60)		1.08 (0.82 to 1.42)	1.19 (0.64 to 2.24)	1.07 (0.69 to 1.66)		1.10 (0.77 to 1.58)		0.98 (0.60 to 1.60)
Low	1.70 (1.17 to 2.47)	1.38 (1.04 to 1.85)		1.50 (1.20 to 1.89)		1.07 (0.82 to 1.40)	1.39 (0.77 to 2.50)	1.54 (1.01 to 2.34)		1.45 (1.03 to 2.04)		0.91 (0.56 to 1.46)
Very low	1.86 (1.30 to 2.68)	2.16 (1.63 to 2.86)		2.00 (1.60 to 2.49)		1.09 (0.84 to 1.41)	2.41 (1.38 to 4.19)	2.07 (1.37 to 3.12)		2.23 (1.61 to 3.09)		1.42 (0.91 to 2.21)
p Value for trend	<0.001	<0.001		<0.001		0.4233	<0.001	<0.001		<0.001		0.0327
*Model 3*												
Very high	1.00	1.00	0.7238	1.00	0.0362	1.00	1.00	1.00	0.8198	1.00	0.9196	1.00
High	0.88 (0.55 to 1.41)	1.12 (0.80 to 1.56)		1.03 (0.79 to 1.35)		0.94 (0.69 to 1.28)	1.11 (0.57 to 2.17)	1.18 (0.75 to 1.85)		1.14 (0.78 to 1.65)		0.91 (0.54 to 1.54)
Moderate	1.16 (0.75 to 1.78)	1.10 (0.79 to 1.54)		1.15 (0.88 to 1.49)		1.08 (0.81 to 1.42)	1.11 (0.55 to 2.23)	1.02 (0.63 to 1.64)		1.03 (0.69 to 1.52)		0.93 (0.57 to 1.52)
Low	1.40 (0.92 to 2.13)	1.44 (1.05 to 1.98)		1.43 (1.11 to 1.84)		1.01 (0.77 to 1.31)	1.26 (0.66 to 2.42)	1.29 (0.81 to 2.05)		1.26 (0.87 to 1.84)		0.85 (0.53 to 1.38)
Very low	1.54 (1.03 to 2.32)	1.87 (1.36 to 2.56)		1.71 (1.34 to 2.19)		1.03 (0.79 to 1.34)	1.86 (1.00 to 3.43)	1.47 (0.92 to 2.34)		1.63 (1.14 to 2.35)		1.29 (0.82 to 2.02)
p Value for trend	0.0024	<0.001		<0.001		0.7503	0.0101	0.0913		0.0028		0.0867

Model 1: adjusted for age.

Model 2: adjusted for age, education, marital status, occupational status, history of CVD and history of cancer.

Model 3: adjusted for age, education, marital status, occupational status, history of CVD, history of cancer, smoking, hypertension, hypercholesterolaemia, diabetes and alcohol intake.

CVD, cardiovascular disease.

The inverse relationship of perceived control with mortality from CVD was stronger than with all-cause mortality and, again, it was considerably stronger in the Czech and Polish cohorts than in Russia (table 3). In the combined Czech and Polish data, the age-adjusted HR for the lowest versus highest control was 3.63 (2.48 to 5.31) in men and 5.20 (2.66 to 10.17) in women. As in the case of all-cause mortality, adjustment in model 2 reduced the estimates by about one-third and in model 3 the effects were about one-half of those seen in model 1. In Russia, there was some suggestion of a weak effect in men in model 1, but there was no association after adjustment for covariates in models 2 and 3 (table 3).

**Table 3:** Association between perceived control and risk of CVD death

	Men						Women					
	Czech Republic	Poland	p Value for interaction (2 countries)	Czech Republic and Poland	p Value for interaction (3 countries)	Russia	Czech Republic	Poland	p Value for interaction (2 countries)	Czech Republic and Poland	p Value for interaction (3 countries)	Russia
	HR (95% CI)	HR (95% CI)		HR (95% CI)		HR (95% CI)	HR (95% CI)	HR (95% CI)		HR (95% CI)		HR (95% CI)
*Model 1*												
Very high	1.00	1.00	0.1033	1.00	0.0004	1.00	1.00	1.00	0.3528	1.00		1.00
High	1.24 (0.67 to 2.32)	1.34 (0.72 to 2.49)		1.32 (0.85 to 2.05)		1.22 (0.81 to 1.84)	2.54 (0.55 to 11.77)	2.37 (1.06 to 5.27)		2.32 (1.14 to 4.71)		0.52 (0.24 to 1.13)
Moderate	1.26 (0.69 to 2.29)	2.32 (1.32 to 4.09)		1.78 (1.18 to 2.69)		1.09 (0.74 to 1.62)	4.03 (0.90 to 17.99)	1.80 (0.77 to 4.22)		2.28 (1.11 to 4.69)		0.80 (0.42 to 1.53)
Low	2.08 (1.18 to 3.64)	3.16 (1.84 to 5.44)		2.65 (1.79 to 3.92)		1.11 (0.76 to 1.61)	5.48 (1.29 to 23.28)	2.09 (0.90 to 4.85)		2.99 (1.49 to 5.99)		0.77 (0.41 to 1.45)
Very low	2.30 (1.34 to 3.97)	5.33 (3.14 to 9.02)		3.63 (2.48 to 5.31)		1.52 (1.07 to 2.16)	7.66 (1.84 to 31.92)	4.59 (2.10 to 10.02)		5.20 (2.66 to 10.17)		1.37 (0.77 to 2.43)
p Value for trend	<0.001	<0.001		<0.001		0.0194	<0.001	<0.001		<0.001		0.0144
*Model 2*												
Very high	1.00	1.00	0.1038	1.00	0.0016	1.00	1.00	1.00	0.4740	1.00		1.00
High	1.00 (0.53 to 1.90)	1.21 (0.65 to 2.25)		1.13 (0.72 to 1.76)		1.17 (0.77 to 1.77)	2.36 (0.50 to 11.10)	2.25 (1.01 to 5.02)		2.17 (1.07 to 4.42)		0.52 (0.24 to 1.14)
Moderate	0.95 (0.51 to 1.75)	1.94 (1.09 to 3.44)		1.44 (0.95 to 2.20)		1.00 (0.68 to 1.49)	2.43 (0.52 to 11.48)	1.60 (0.68 to 3.77)		1.71 (0.81 to 3.59)		0.73 (0.38 to 1.40)
Low	1.55 (0.87 to 2.75)	2.42 (1.40 to 4.20)		2.02 (1.36 to 3.00)		1.01 (0.69 to 1.46)	4.21 (0.98 to 18.08)	1.86 (0.80 to 4.34)		2.44 (1.20 to 4.93)		0.68 (0.36 to 1.28)
Very low	1.48 (0.84 to 2.59)	4.04 (2.36 to 6.91)		2.57 (1.74 to 3.79)		1.19 (0.83 to 1.70)	5.31 (1.26 to 22.35)	3.86 (1.75 to 8.55)		4.06 (2.06 to 8.02)		1.15 (0.65 to 2.05)
p Value for trend	0.0380	<0.001		<0.001		0.4276	0.0012	0.0013		<0.001		0.0841
*Model 3*												
Very high	1.00	1.00	0.2302	1.00	0.0063	1.00	1.00	1.00	0.6844	1.00		1.00
High	0.77 (0.37 to 1.61)	1.22 (0.60 to 2.49)		1.04 (0.62 to 1.72)		1.14 (0.75 to 1.73)	2.92 (0.34 to 25.07)	3.49 (1.18 to 10.35)		3.15 (1.20 to 8.31)		0.50 (0.24 to 1.14)
Moderate	0.87 (0.44 to 1.73)	1.78 (0.92 to 3.45)		1.35 (0.84 to 2.18)		0.98 (0.66 to 1.47)	4.23 (0.51 to 35.25)	2.93 (0.95 to 9.05)		3.03 (1.13 to 8.14)		0.66 (0.34 to 1.28)
Low	1.18 (0.61 to 2.26)	2.60 (1.39 to 4.84)		1.93 (1.24 to 3.02)		0.94 (0.64 to 1.38)	6.00 (0.78 to 46.40)	3.12 (1.01 to 9.68)		3.71 (1.41 to 9.75)		0.64 (0.34 to 1.22)
Very low	1.20 (0.64 to 2.25)	3.83 (2.07 to 7.06)		2.31 (1.48 to 3.59)		1.11 (0.77 to 1.59)	7.26 (0.96 to 54.67)	5.17 (1.74 to 15.39)		5.50 (2.14 to 14.13)		0.99 (0.55 to 1.77)
p Value for trend	0.2066	<0.001		<0.001		0.7488	0.0069	0.0057		<0.001		0.2244

Model 1: adjusted for age.

Model 2: adjusted for age, education, marital status, occupational status and history of CVD.

Model 3: adjusted for age, education, marital status, occupational status, history of CVD, smoking, hypertension, hypercholesterolaemia, diabetes and alcohol intake.

CVD, cardiovascular disease.

In additional sensitivity analyses, we investigated the association of mortality with perceived control scale (1) with the three health-related items removed from the control scale, (2) with additional adjustment for presence of other chronic conditions at baseline, and (3) after ignoring deaths within the first 2 years of follow-up. The results were robust to model specification and remained similar to the main analyses presented above.

## Discussion

In this large prospective study in three Eastern European populations, low perceived control was associated with increased risk of total and CVD mortality in two of the three cohorts. In the Czech and Polish cohorts, the associations were statistically significant, graded and, although attenuated, they persisted after adjustment for a range of covariates. In contrast, the associations in Russian men and women were weak and did not survive multivariable adjustment.

The results from the Czech and Polish cohorts confirmed earlier findings from the Polish cohort with a shorter follow-up period^27^ and are consistent with results reported in the USA and Western Europe.^19–21^ In Russia, in contrast, we did not find an association of perceived control with total or CVD mortality. The latter finding is contrasting with previous reports on associations between perceived control and self-rated health from cross-sectional studies.^26^ ^28^ ^29^ However, this might reflect the differences in the study design and the nature of outcome variable.

The mean perceived control scores in this study were similar across the three cohorts. This was not entirely expected, since the New Democracy Barometer surveys, which used identical questions, found lower levels of perceived control (and higher mortality) in Russian than in other postcommunist countries,^28^ and this led to speculation of its potential role in poor health in Russia.^26^ On the other hand, another multicountry survey suggested relatively high level of perceived control in Russian adults.^30^ We are not certain whether such differences between studies are due to differences in methodology, differences in historical periods when the surveys were conducted or other factors.

The lack of association between perceived control and mortality in the Russian cohort is puzzling, as it may indicate that control is not a universal predictor of mortality. The Russian sample had sufficient statistical power to demonstrate association between perceived control and mortality of similar magnitude as observed in the Czech and Polish cohorts. On the other hand, the lack of association in Russia may be due to measurement issues. Although the questionnaires were repeatedly translated from English and back-translated to each language and checked for inconsistencies, it is possible that the words used in the Russian translation do not tap into the concept of control as understood by Russians. There is some indication that perceived control may have a different meaning in Russia.^31^ In addition to the semantics of the Russian language, the sociocultural specificity of Russia should also be considered, as Russian society represents a unique combination of historically determined social and cultural characteristics that might influence perception of control of individuals. There is some evidence that individuals from collectivistic cultures have a strong belief in external sources of control.^32–34^ Russia represents rather a collectivistic culture;^35^ historically, Russian society had highly centralised structure of power followed by high obedience to authorities, coexisting with authoritarian family practices.^36^ ^37^ It has been pointed out that the phrases used in Russian and the general tone of the language are often characterised by lack of enhancement of the individual as controller of events and present humans as not controlling their lives and with some tendency to submissiveness.^38^ In contrast, for Czechs and especially for Poles personal freedom was always highly valued, and it is likely that these countries represent more individualistic cultures.^38^ These cultural factors might have affected both the assessment of perceived control and the relations between perceived control and health.

The inverse association of perceived control with total and CVD mortality found in the Czech and Polish cohorts appears plausible. Although the mechanism linking perceived control with health are not fully understood, perceived control was found to be related with important psychological and physiological processes. There is evidence on the role of perceived control as longitudinal moderation of late-life stressors on depressive symptoms^39^ and relations with anxiety disorders,^40^ neural responses to setbacks,^41^ vasovagal response,^42^ imbalance of immunological system^43^ and influence of genetic variance in physical health.^44^ The dose–response relationship in these two cohorts further supports the notion that the association is genuine.

In addition to the crucial issue of the comparability of the assessment of perceived control across different countries, there are several other limitations.

First, response rates in all three cohorts were modest (between 55% and 62%). However, as the rates were comparable in all three centres, it is unlikely that the differences in the associations between Poland, Czech Republic and Russia could have resulted from differences in response rates. Several studies showed that declines in participation rates in epidemiological studies in the last decades do not necessarily affect the estimates of examined associations^45^ ^46^ but it is likely that low participation had an impact on representativeness of the sample. There is some evidence that the samples suffered from a health selection bias as non-respondents had higher risk of death compared with study participants;^47^ the relationship between perceived control and mortality was therefore investigated in a healthier part of the original population sample.

In relation to potential measurement problems, it might be argued that different scales of perceived control could produce different results, although a fair degree of stability across time and situations for measures of perceived control has been reported.^11^ Furthermore, adjustment for socioeconomic status had some limitations. Socioeconomic status is determined also by many other characteristics, which were not measured. Similar to numerous other studies, we used education and occupational status only in the analysis.

Second, although the number of exclusions from the analysis due to availability of follow-up and perceived control data is low, a considerable number of missing data in biochemical tests and measurements reduced the number of participants included to the final analysis (model 3). However, the sensitivity analysis restricted to participants with all complete records in models 1 and 2 was also performed and it did not change the results substantially (data not shown).

Third, participants were sampled from urban populations and the cohorts cannot be regarded as representatives of the whole countries. Although the analysis of Polish data suggested that the Polish HAPIEE sample was similar to other large Polish cities in terms of marital status, occupational status, prevalence of main CVD risk factors and SCORE (Systematic COronary Risk Evaluation) risk assessment,^48^ extrapolations from our study to the broader population must be made with cautions.

Fourth, the possibility of reverse causation cannot be excluded in observational studies. Participants in poor health may feel and report lower control. Although our sensitivity analyses appeared robust to omitting health items from the control scale, adjustment for baseline health and excluding deaths in the first 2 years of follow-up, reverse causation cannot be entirely ruled out.

Fifth, the multivariable model 3 may have led to an overadjustment, since some of the covariates could be mediators (eg, smoking or alcohol consumption), as low perceived control may lead to unhealthy behaviours which, in turn, result in increased mortality risk.

On the other hand, our study has important strengths. First, this is the first report on the relationship between perceived control and mortality in the Central and Eastern European region which differs substantially from the West in the historical, social and economic context. Second, this is a prospective study, which minimises the potential for reverse causation. Finally, as we collected a wide range of social, behavioural and biological risk factors, we were able to control for a number of variables and to minimise the risk of confounding.

In conclusion, results observed in two of our three cohorts support the hypothesis and previous reports that low perceived control has negative effect on health and on CVD in particular. However, the lack of the association in the Russian cohort raises questions about the universal applicability of the concept of perceived control, and requires further clarification of the concept and its measurement in more collectivistic societies.

**What is already known on this subject?**

- It has been hypothesised that perceived control (often described as individuals' belief in own capability to change their behaviour, influence the environment and attain the desired action outcomes) is associated with reduced chronic psychosocial stress.
- There is some evidence from stable, affluent and relatively individualistic societies of Western Europe and the USA that perceived control is associated with lower mortality from all causes and cardiovascular diseases. It is less clear whether the same association exists in other societies.

**What this study adds?**

- The HAPIEE study is the largest prospective study on the association between psychosocial factors and cardiovascular diseases in Central and Eastern Europe. These countries have undergone dramatic social and economic changes after the fall of communism in 1989.
- The present analysis found inconsistent results. The inverse association between perceived control and all-cause and cardiovascular mortality was confirmed in Czech Republic and Poland but not in Russia. This lack of the association in the Russian cohort raises questions about the applicability of the concept of perceived control to more collectivistic societies.
